# Adherence to a digital therapeutic mediates the relationship between momentary self-regulation and health risk behaviors

**DOI:** 10.3389/fdgth.2025.1467772

**Published:** 2025-02-04

**Authors:** Enzo G. Plaitano, Daniel McNeish, Sophia M. Bartels, Kathleen Bell, Jesse Dallery, Michael Grabinski, Michaela Kiernan, Hannah A. Lavoie, Shea M. Lemley, Michael R. Lowe, David P. MacKinnon, Stephen A. Metcalf, Lisa Onken, Judith J. Prochaska, Cady Lauren Sand, Emily A. Scherer, Luke E. Stoeckel, Haiyi Xie, Lisa A. Marsch

**Affiliations:** ^1^ Center for Technology and Behavioral Health, Geisel School of Medicine, Dartmouth College, Lebanon, NH, United States; ^2^ The Dartmouth Institute for Health Policy and Clinical Practice, Geisel School of Medicine, Dartmouth College, Hanover, NH, United States; ^3^ Department of Psychology, Arizona State University, Tempe, AZ, United States; ^4^ Department of Health Behavior, University of North Carolina at Chapel Hill, Chapel Hill, NC, United States; ^5^ Department of Psychology, University of Florida, Gainesville, FL, United States; ^6^ Stanford Prevention Research Center, Stanford University, Stanford, CA, United States; ^7^ Department of Health Education and Behavior, University of Florida, Gainesville, FL, United States; ^8^ Department of Psychological and Brain Sciences, Drexel University, Philadelphia, PA, United States; ^9^ Department of Public Health and Primary Care, University of Cambridge, Cambridge, United Kingdom; ^10^ National Institute on Aging, National Institutes of Health, Bethesda, MD, United States; ^11^ Apple Inc., Cupertino, CA, United States

**Keywords:** digital therapeutic, momentary self-regulation, health risk behavior, smoking, binge eating disorder, obesity, ecological momentary assessment (EMA)

## Abstract

**Introduction:**

Smoking, obesity, and insufficient physical activity are modifiable health risk behaviors. Self-regulation is one fundamental behavior change mechanism often incorporated within digital therapeutics as it varies momentarily across time and contexts and may play a causal role in improving these health behaviors. However, the role of momentary self-regulation in achieving behavior change has been infrequently examined. Using a novel momentary self-regulation scale, this study examined how targeting self-regulation through a digital therapeutic impacts adherence to the therapeutic and two different health risk behavioral outcomes.

**Methods:**

This prospective interventional study included momentary data for 28 days from 50 participants with obesity and binge eating disorder and 50 participants who smoked regularly. An evidence-based digital therapeutic, called Laddr™, provided self-regulation behavior change tools. Participants reported on their momentary self-regulation via ecological momentary assessments and health risk behaviors were measured as steps taken from a physical activity tracker and breathalyzed carbon monoxide. Medical regimen adherence was assessed as daily Laddr usage. Bayesian dynamic mediation models were used to examine moment-to-moment mediation effects between momentary self-regulation subscales, medical regimen adherence, and behavioral outcomes.

**Results:**

In the binge eating disorder sample, the perseverance [*β*_1_ = 0.17, 95% CI = (0.06, 0.45)] and emotion regulation [*β*_1_ = 0.12, 95% CI = (0.03, 0.27)] targets of momentary self-regulation positively predicted Laddr adherence on the following day, and higher Laddr adherence was subsequently a positive predictor of steps taken the same day for both perseverance [*β*_2_ = 0.335, 95% CI = (0.030, 0.717)] and emotion regulation [*β*_2_ = 0.389, 95% CI = (0.080, 0.738)]. In the smoking sample, the perseverance target of momentary self-regulation positively predicted Laddr adherence on the following day [*β* = 0.91, 95% CI = (0.60, 1.24)]. However, higher Laddr adherence was not a predictor of CO values on the same day [*β*_2_ = −0.09, 95% CI = (−0.24, 0.09)].

**Conclusions:**

This study provides evidence that a digital therapeutic targeting self-regulation can modify the relationships between momentary self-regulation, medical regimen adherence, and behavioral health outcomes. Together, this work demonstrated the ability to digitally assess the transdiagnostic mediating effect of momentary self-regulation on medical regimen adherence and pro-health behavioral outcomes.

**Clinical Trial Registration:**

ClinicalTrials.gov, identifier (NCT03774433).

## Introduction

Health risk behaviors, such as smoking, obesity, and insufficient physical activity lead to the development of chronic diseases and early mortality (1, 2). As much as 40% of premature deaths in the United States and 60% of all deaths in Europe are attributed to health risk behaviors (3, 4). In low- and middle-income countries, deaths from noncommunicable, health behavior-related diseases are projected to rise substantially from 30 million to 41.8 million by the year 2030 (5). Predictive models suggest that by 2030, half of the adults in the United States will experience obesity with a prevalence above 50% in 29 states (6). Similar models predicted that 110,000 premature deaths could be prevented each year in the United States if adults engaged in ten more minutes of daily moderate-to-vigorous physical activity (7). While smoking prevalence has decreased as much as 38% from 1990 to 2020 worldwide, a total of 50.9 million adults in the United States still reported current use of tobacco in 2022 with 80.5% of those individuals reporting smoking tobacco products (8, 9).

While numerous pharmacotherapies and behavioral interventions seek to target health risk behaviors, non-adherence to prescribed medical regimens is ubiquitous and associated with worse health outcomes, higher disease prevalence, and increased healthcare costs across multiple chronic diseases (10, 11). This lack of adherence contributes to approximately 125,000 avoidable deaths and as much as 69% of hospitalizations every year in the United States (12). A meta-analysis of over 500 studies found an average rate of non-adherence to prescribed medical treatment of 24.8%, which equates to over 188 million medical visits where patients do not follow prescribed medical advice (13). Specifically, as much as 30% of prescription medications are never filled by patients, and over 50% of patients discontinue their prescription medications without consultation with their physician (14–16). Medication nonadherence can result from patient-related barriers such as lack of motivation, fear of side effects, cultural factors, or treatment-related barriers such as difficulty accessing pharmacies, high costs of brand name medications, poor patient-provider communication, or too little time to fill prescriptions (17). Targeting health risk behaviors can have an immense impact on improving chronic disease outcomes and reducing excessive healthcare utilization in people non-adherent to medical regimens (18–21).

One fundamental behavior change mechanism across different types of health behavior is self-regulation. Self-regulation is defined as a person's ability to control motivations, cognition, and emotions to avoid immediate gratification and achieve their long-term goals (22). Multiple studies have identified the role of self-regulation as a potential causal mechanism to promote better health behavior and have identified deficient regulation as a causal mechanism in health risk behaviors, including cigarette smoking, insufficient physical activity, and binge eating (23–31). Until recently, self-regulation has typically been studied as a static trait-level characteristic, but it is increasingly considered a dynamic process with intraindividual variability across time and contexts (32, 33). This malleable process suggests that both internal contexts, such as withdrawal or craving, and external contexts, such as environmental factors, may impact an individual's self-regulation in the moment.

Recent advancements in digital technology have created new opportunities to assess and modify self-regulation in large populations and in real-time. Specifically, the growing field of digital health uses tools such as smartphone applications and wearable devices to deliver targeted health interventions (34). Digital health interventions, typically referred to as digital therapeutics, can enable people to better monitor their health status, change behaviors, understand competing health priorities, make informed treatment decisions, manage medications, and improve patient-provider communication (35). These outcomes are achieved through complex interventional components, such as health monitoring tools, psychoeducation, decision aids, and evidence-based cognitive behavioral therapies and/or mindfulness interventions traditionally delivered only through in-person consultation (35).

Digital therapeutics offer the promise to be wide reaching, given that 85% of households in the United States have access to the internet, 84% own a smartphone, and 92% own a computer (36). Importantly, rural communities across the United States with less access to traditional healthcare still often have high rates of internet and computer access (37). Further, the majority of people around the world own a mobile phone with a prevalence as high as 83% in countries with emerging economies (38). Given this prevalence, digital health interventions have been tested in high-, low- and middle-income countries and have been found to improve adherence to medical regimens, appointment attendance, and gathering of health data to enhance clinical outcomes for a wide array of populations (39–43). Digital health interventions have been shown to lead to outcomes comparable to or better than in-person clinician-delivered interventions and “just in time” support during everyday activities or when clinicians are not available (44–49). The worldwide access to digital technologies provides the opportunity to broadly assess and promote self-regulation at a population level.

While numerous interventions have utilized digital health technology to increase medical regimen adherence and pro-health behavior, most of this work has been siloed, focusing on only one disease or disorder at a time (50). Additionally, the proposed mechanisms underlying these behavior change interventions are infrequently systematically examined. In response, the National Institutes of Health (NIH) Science of Behavior Change (SOBC) initiative was developed to overcome these silos and systematically examine the mechanisms of behavior change interventions using an “experimental medicine” approach (51). Particularly, the program sought to (1) use basic science to investigate mechanisms of behavior change across different health behaviors, (2) go beyond clinical endpoints to find common predictable targets of health behaviors, and (3) incorporate these mechanistically informed targets from basic science to modify clinical endpoints through replicable and scalable behavioral health interventions (51). Within the framework of the SOBC initiative, understanding the role of self-regulation as a key target across different health behaviors can help develop “precision medicine” approaches for more efficient, cost-effective, and patient-centered care across numerous populations. In response, our interdisciplinary team has been using the SOBC framework to study the role of self-regulation as a putative behavior change target across different health risk behaviors with the goal of developing efficacious and scalable digital health interventions.

To better understand these dynamic self-regulatory processes, we previously developed and validated a novel momentary self-regulation scale in a large sample of over 500 nationally recruited adults (52). Given the advantages of digital health platforms, this scale was administered via ecological momentary assessments (EMA) on mobile devices to assess momentary self-regulation in a nonlaboratory, naturalistic setting. As detailed in our previous publication, the scale comprises four factors of self-regulation, including momentary perseverance, sensation seeking, emotion regulation, and mindfulness (52). Initial testing of the selected items showed strong construct and predictive validity for health risk behaviors and suggests this novel momentary self-regulation scale can be successfully administered via mobile devices as ecological momentary assessments (EMAs) (52).

In another study, a novel digital health application called Laddr™ distributed this novel scale through EMA (33). Laddr is an evidence-based behavior change digital therapeutic delivered through an interactive, self-directed smartphone application and has been studied across numerous health risk behaviors, including smoking, binge eating, illicit drug use, alcohol use, and mental health concerns such as depression, anxiety, and panic disorder (39, 40, 53–55). Laddr includes self-regulation monitoring and behavior change tools to help people solve problems, conceptualize obstacles, and develop skills to overcome their health risk behaviors and maintain motivation to behavior change. The EMA data from this interventional platform were used to examine if putative targets of momentary self-regulation changed in different real-world environmental contexts and if the Laddr digital therapeutic modified the association between momentary self-regulation and these contexts (33). This prior work was conducted with 104 people who smoke regularly and 81 people with obesity/overweight and binge eating disorder (BED). Smoking and BED are two exemplar medical concerns in which the health behaviors may contribute to decreased medical regimen adherence (1). Results from this prior study suggest it is possible to trans-diagnostically measure momentary self-regulation and study the effect of a momentary mobile intervention on self-regulation in a non-laboratory setting (33). Specifically, this study suggests that momentary self-regulation may have intraindividual variability depending on real-world internal (e.g., negative affect) and external contexts (e.g., smelling smoke) applied to these different health risk behaviors (33). Smoking and overeating may decrease momentary self-regulation while the Laddr digital therapeutic may increase momentary self-regulation thus underscoring the malleability of self-regulation (33). This work was important in informing our next step, reported in the present manuscript, of studying the mediating effect of momentary self-regulation on health behavior, defined as adherence to medical regimens, and health outcomes related to smoking and obesity.

The primary objective of the current study was to examine how targeting self-regulation through the Laddr digital therapeutic engages putative behavior change targets in two defined subgroups of interest: (1) people who smoke regularly and (2) people with obesity/overweight status meeting criteria for BED. This mobile digital health intervention integrates both novel momentary self-regulation assessments and behavior change tools on a modifiable platform that allows the content to be quickly adapted as needed to better impact targets. Specifically, this study examined the degree to which Laddr engages putative self-regulatory targets of behavior change in people with obesity/overweight and BED and people who smoke regularly to impact adherence to the Laddr intervention as well as health behaviors of smoking and physical activity among the participant samples, respectively. We hypothesized that across both people with obesity/overweight and BED and people who smoke regularly (1) self-regulation will predict adherence to treatment using the Laddr intervention on the following day, (2) treatment adherence will then predict the objective behavioral health outcome through either the number of steps taken or the carbon monoxide sample value, and (3) treatment adherence and the behavioral outcome will both predict self-regulation at the next measurement. A key goal of this line of research is to demonstrate mechanisms of behavior change that are necessary to target to then help people achieve meaningful clinical endpoints.

## Materials and methods

### Study design

The present study was a 28-day prospective interventional study designed to target self-regulation to impact health behaviors and health outcomes. This study protocol was approved by the Dartmouth College Institutional Review Board, monitored by a Dartmouth College Data and Safety Monitoring Board, and the study protocol was preregistered on ClinicalTrials.gov (Identifier: NCT03774433).

### Participants

#### Target population

This study recruited two distinct samples of participants: (1) people with obesity/overweight that met the screening criteria for BED and (2) people who smoke regularly. These are two exemplar groups of people where engaging in health risk behaviors can contribute to negative health outcomes (1, 2).

#### Target sample size

The total target sample size was 154 participants (77 people with obesity/overweight and BED and 77 people who smoke regularly) recruited nationally from the United States. The target of 77 participants from each group was determined to reach a final sample size of at least 50 participants in each group with adequate EMA participation of at least 10% of responses over the duration of the 28-day study (≥12 out of 112 total EMAs). These values were derived from power calculations of the percentage of participants who completed at least 10% of their EMAs in our earlier study examining how different contexts may impact momentary self-regulation (33). The maximum number of observations in the momentary dataset would be 8,624 with 77 participants in each group and 4 EMA responses per day for 28 days. The momentary dataset would be closer to 5,821 with a 10% study drop-out and 75% EMA completion rate. With this sample size, there would be adequate power to detect momentary relationships between contextual factors and momentary self-regulation.

#### Overall sample eligibility

All participants had to reside in the United States, be 18–50 years old, provide informed consent in English, have access to a computer in a comfortable setting conducive to providing sensitive information, and use a smartphone with an operating system compatible with Laddr. Exclusion criteria were: enrolled in our earlier study examining putative targets of momentary self-regulation and associations with Laddr, current diagnosis of a substance use disorder other than nicotine use disorder, use of prescription pain medications, use of medications for smoking (not including ​​nicotine replacement therapy), use of medications for weight loss, pregnancy or plans to become pregnant in the next 3 months, lifetime history of a mental disorder specifically determined to be due to a medical condition or classified as a psychotic disorder (e.g., schizophrenia, schizoaffective disorder, or bipolar disorder, but not including depression), medical history of weight-loss surgery, and nighttime shift work or obstructive sleep apnea. These variables were all potential confounders that may be related to momentary self-regulation, Laddr adherence, or the outcomes of interest. The following study inclusion criteria intended to have participants in the two groups be non-overlapping samples so that we could examine the hypothesized relations across these two distinct health risk behaviors.

#### Binge eating sample eligibility

In addition to the overall study eligibility criteria, the BED sample had to have a BMI ≥ 27 kg/m^2^ and ≤45 kg/m^2^, screen positive for binge eating behavior based on the QEWP-5, currently non-smoking (defined as no cigarettes in past 12 months), confirmed interest in an eating intervention, and use a smartphone compatible with the Fitbit application (Fitbit Flex 2™). Additional exclusion criteria were: compensatory behavior (e.g., purging, excessive exercise, fasting) at a frequency of once or more per week (less than once per week, on average, was not considered exclusionary), weight loss >10 pounds in the past six months, currently in a weight-loss program (excluding online/mobile app weight-loss programs), current use of a special diet for a serious health condition, current therapy with a clinician for binge eating, and known nickel allergy because the Fitbit band contained nickel.

#### Smoking sample eligibility

In addition to the overall study eligibility criteria, the smoking sample had to smoke five or more cigarettes/day for the past year, have a BMI ≥ 17 kg/m^2^ and <27 kg/m^2^, confirmed interest in a smoking cessation intervention, and use a smartphone compatible with the iCO Smokerlyzer device (iCO™ Smokerlyzer®, Bedfont® Scientific Ltd.). Additional exclusion criteria were: screening positive for binge eating behavior according to the Questionnaire on Eating and Weight Patterns-5 (QEWP-5), and current therapy with a clinician for smoking and/or BED. Participants in the smoking sample were allowed to use nicotine replacement medications, but could not use any other prescription medications that may promote smoking cessation (e.g., bupropion, varenicline).

### Recruitment

Participants were recruited online through social media platforms (Craigslist, Facebook, Instagram, and Google AdWords), which proved successful in the earlier study examining putative targets of momentary self-regulation and associations with Laddr (33). After engaging with an online study ad, participants were directed to an online eligibility screening questionnaire on the Dartmouth Research Electronic Data Capture (REDCap) platform (56, 57). Participants who met eligibility criteria on the online screening questionnaire were then verified via phone by study staff. Participants who provided informed consent were scheduled for the study and mailed study supplies with no in-person visit necessary. To obtain a diverse cohort representative of behavioral targets of interest, we developed and utilized demographically targeted ads and enrolled participants based on gender, race, and ethnicity. Recruitment and data collection occurred from February 27, 2019, through June 29, 2020.

### Laddr intervention

The Laddr digital therapeutic is a transdisciplinary evidence-based behavior change intervention delivered via an interactive, self-directed mobile platform (58). Laddr is unique in that it uses science-based therapeutic tools for behavior change across a wide range of disorders and behaviors based on their specific goals and needs. The validated tools within Laddr have been informed by over 20 NIH-supported randomized trials and includes behavior change tools for multiple health risk behaviors, including smoking, binge eating, substance use, alcohol use, and mental health problems such as depression, anxiety, and panic disorder (39, 40, 53–55). This platform has various guides that provide specific self-regulation monitoring and behavior change tools to solve problems, overcome obstacles, and develop skills for maintenance of these learned behaviors. Information about binge eating and smoking are embedded within the guides to help users create informed personalized goals regarding their health risk behaviors. For example, the smoking guides included information on first recognizing and learning about smoking cues. Then, participants completed problem solving activities to help employ healthier solutions. Suggested healthier solutions included performing breathing exercises or going outside for a walk to remove themselves from the cues. Additionally, Laddr allows users to enter their health data into the platform, such as inputting daily Fitbit activity (BED sample) and daily carbon dioxide (CO) readings (smoking sample) to track progress over time.

### Measures

#### Surveys

All participants were asked to complete a battery of baseline surveys at the beginning of their 28-day study period. These surveys were expected to take approximately 1.5–2 h at each time point. The baseline survey measured demographics and socioeconomic status (e.g., age, sex, ethnicity, race, education), physical health characteristics (e.g., height and weight for BMI calculation), and smoking and binge eating-related information (e.g., cigarettes smoked per day or ever in a weight loss program). Participants also completed a battery of 17 questionnaires on self-regulation, described in detail in our earlier study examining putative targets of momentary self-regulation (33). Additionally, participants in the BED sample completed questions adapted from the QEWP-5 regarding past month eating habits to screen for BED behaviors at baseline (59). In the smoking sample, participants completed the Fagerström Test for Nicotine Dependence (60). Participants were asked to abstain from smoking (smoking sample only) and eating (BED only) for three hours prior to the start of the baseline assessments. Regardless, participants were asked questions assessing their last consumption of nicotine (smoking sample only) or food (BED sample only) prior to starting the baseline survey.

#### Putative targets

During the study period, participants completed items related to self-regulation and context. The items included the momentary self-regulation questionnaire used in our earlier study examining putative targets of momentary self-regulation, which was delivered through mobile EMA (33, 52). This 12-item scale assesses four factors of self-regulation in the moment: (1) perseverance, (2) sensation seeking, (3) emotion regulation, and (4) mindfulness (52). Briefly, momentary perseverance includes items on goal setting, tracking, and continuing to focus on them until they are finished. Momentary sensation seeking includes items on taking and enjoying risks (reverse coded). Momentary emotion regulation includes items on effectively managing and responding to emotional experiences. Lastly, momentary mindfulness includes items of attention and mindfulness related to a specific task (52). These items had strong construct and predictive validity and intra- and interindividual variability for health risk behaviors in our previous studies and proved that this novel momentary self-regulation scale can be successfully administered through mobile devices (52).

The EMAs also assessed momentary context measures. These measures were described extensively in earlier work and included items such as mood (positive and negative affect, stress, and tiredness), companionship (whether they were alone or with others), location (e.g., home, friend or family member's house, work), and sample-specific temptations to smoke or binge eat (33). EMAs were delivered through Laddr four times daily at random times within time windows at least one hour apart (e.g., 8–11:30 AM, 11:30 AM–3 PM, 3–6:30 PM, 6:30–10 PM) based on participants' self-reported sleeping and waking hours in their local time zones. EMAs also included questions about the health risk behaviors of interest depending on the sample, including access to cigarettes or food, urge to smoke or binge eat, and if they smelled smoke or food. Based on the average EMA completion time in the previous study, we expected each EMA to take less than five minutes (33).

#### Medical regimen adherence

Participants were asked to use Laddr daily to update their progress towards self-regulatory goals and complete various activities on the smartphone application that were expected to improve their self-regulation. As part of this adherence to the intervention, participants were recommended priority therapy guides, located directly within the Laddr application, such as binge eating guides for the BED sample and smoking guides for the smoking sample, but could also choose to engage with other guides, such as those for depression, anxiety, and substance use. Participants were also instructed to enter their daily Fitbit activity (BED sample) and daily CO readings (smoking sample) into the Laddr platform to quantitatively track progress over time. The outcome for medical regimen adherence was the use of Laddr each day for at least 5 min over the 28 consecutive day study period. This was assessed through examining Laddr usage data for each day of the study and coded a binary adherence variable (yes or no). Participants who spent at least 5 min in the app on each day were adherent.

#### Health behavior outcomes

Participants were asked to use devices to measure health risk behaviors and collect putative mechanisms data. The BED sample was provided with a physical activity tracking device, the Fitbit Flex 2™, and downloaded the Fitbit app on their smartphone using study credentials to track steps, distance, calories burned, active minutes, hourly activity, and stationary time. Participants were asked to wear the activity tracker for at least 12 h per day and use a changing criterion design to set stepped goals. In this design, participants set a 7-day activity criterion goal and were encouraged to increase their criterion goals in each subsequent 7-day block if they achieve the goal on at least 4 out of the 7 days (61). Physical activity data were collected by the study team in near-real-time through Fitabase, which is a secure research data management platform designed specifically for Fitbit device use. Activity data were uploaded from the Fitbit device to Fitabase every 15 min if a wireless connection was maintained or all at once when a connection to the device was re-established.

The smoking sample was provided with a breath CO meter, the iCO™ Smokerlyzer® (Bedfont® Scientific Ltd.), and downloaded the Smokerlyzer app on their smartphones to provide one CO sample in the same time window each day. During this procedure, participants exhaled into the mouthpiece of the CO meter and recorded themselves via their device camera. Specifically, this device measured CO to CO_2_ conversion over a catalytically active electrode in parts per million (ppm) (62). We examined if participants in the smoking sample initially reduced their CO levels from baseline levels and then achieved smoking abstinence which equates to a CO measure less than 6 ppm (63). Breath CO has a half-life of about 4–6 h, which makes it an ideal, more objective measurement for this study (64, 65). This procedure to measure breathalyzed CO as an outcome of digital health interventions has been utilized by our team in numerous other studies (55, 66–68).

### Retention

#### Compensation

Participants were compensated up to $250 in a single payment at the end of their study period via an Amazon gift card or a paper check after returning any borrowed research equipment. This included completion of surveys ($21 for all baseline and $21 for all follow-up surveys), EMAs ($0.50 per EMA with 4 EMAs per day over 28 days, up to $56 total; $10 bonus per week for completing minimum of 25 EMAs over 4 weeks, up to $40 total), Laddr activities ($2 per day for at least 5 min of daily engagement over 28 days, up to $56 total), and wearing the Fitbit wrist sensor daily (BED sample) or using the CO monitor daily (smoking sample) and inputting these values into Laddr ($2 per day over 28 days, up to $56 total). Participants were still compensated even if they did not return the borrowed research equipment, however, their total payment was reduced by up to $50.

#### Compliance and reminders

The study team monitored data quality on a daily basis. EMA completion rates, Laddr app usage, and input CO values were downloaded and examined daily, while steps were tracked daily in Fitabase. Poor compliance regarding any data source was addressed through reminders sent to the participant's smartphone through both text message or Laddr app notifications and through their email addresses.

### Psychometric analyses

In the Bayesian structural equation models used in this mediation analysis, described later in the methods, it is important to first establish invariance of measures across both time and people to (a) ensure that the meaning of the items does not drift over time and (b) that the items are interpreted similarly for different people in the sample (69–72). Otherwise, changes in the measurement process might be conflated with changes in the underlying constructs. Cross-classified factor analysis was used to assess between-person and between-time invariance in EMA data (73, 74). Approximate invariance was achieved if the variance in the measurement model parameters was small (75).

In the BED sample, the perseverance items were modeled as continuous and items from all other scales were modeled as categorical with a probit link, so loading means are interpreted as regression coefficients for perseverance but item discriminations for all other scales (76). This was done because only perseverance had approximately symmetric distributions. None of the scales had large between-time variability, meaning that the scales were stable across time points. However, all scales had some between-person variability, meaning that different people did not always interpret the scales in the same way. For perseverance, one item loaded weaker than the other two items, so the scale was modeled as a latent variable because a sum score would equally weigh all items (74). The full dynamic mediation model for perseverance included between-person random effects on the item parameters to account for possible differences in scale interpretation. For both the emotion regulation and mindfulness scales, one item was highly discriminating on each scale, but all items contributed to each latent variable. Therefore, the mediation models of emotion regulation and mindfulness included between-person random effects on all item parameters. Similar to the CO readings, steps taken were rescaled by dividing the raw value by 1,000 (e.g., a one-unit change corresponds to 1,000 steps) to improve the estimation by placing all variables on the same order of magnitude.

In the smoking sample, none of the scales had large between-time variability in item parameters but all the scales had between-person differences. Perseverance was modeled as a latent variable with all three indicators and included between-person random effects. The emotion regulation item only had two of the three categories endorsed with regularity, so the item was treated as dichotomous. Similarly, the mindfulness item rarely had the most extreme response selected so the two most extreme categories were collapsed into one and the item was treated as four categories instead of five. Additionally, the quantitative CO readings were rescaled on a 0–10 scale rather than 0–100 to make the scales in the analyses more congruent as well (e.g., 12 ppm on 0–100 transformed to 1.2 on 1–10).

Supplementary Table S1 shows the results from the cross-classified factor analysis for each of the three self-regulation scales and the average loading of each item across all people and measurement occasions, the variance of the loadings across people, the variance of the loadings across time, the variance of the intercepts/thresholds across people, and the variance of the intercepts/thresholds across time (74).

### Statistical analyses

For the statistical models, we hypothesized that (1) participants' self-regulation will predict whether they will adhere to treatment using the Laddr intervention on the following day across both people with obesity/overweight and BED and people who smoke regularly. We hypothesized that (2) treatment adherence will then predict the objective behavioral health outcome through either the number of steps taken or the CO sample value. Lastly, we hypothesized that (3) treatment adherence and the behavioral outcome then predict self-regulation at the next measurement occasion. The models examined whether self-regulation affected health behavior through treatment adherence.

Data analysis was performed using Mplus version 8.3 with Bayesian Markov chain Monte Carlo estimation with a Gibbs sampler with two chains (77). Bayesian dynamic mediation models were used to examine moment-to-moment mediation effects between the momentary self-regulation subscales, medical regimen adherence (use of Laddr at least daily), and number of steps taken for BED sample or CO samples for the smoking sample (78, 79). This model allowed for maximized use of the repeated measures collected through EMA to examine changes in means over time and to inspect fluctuation within a person over time (Supplementary Figure S1). To overcome unequal intervals between measurements inherent in EMA modeling, a Kalman filter was applied to naturally accommodate data with unequal time intervals between measurements with a maximum interval of four hours (78–80).

Dynamic mediation models were fit for both the BED and smoking samples. These included one model for each permutation of three self-regulation scales (perseverance, emotion regulation, mindfulness) and the two behavioral outcomes (CO samples or steps taken). The sensation seeking scale was not included in the analysis. Psychometric analyses indicated that the scale items did not represent a single construct in the sample, thus the decision was made not to model the sensation seeking items as one construct like the other self-regulation scales, which would be inappropriate.

Because Bayesian estimation was used, there are no *p*-values or significance tests, and inference is based upon whether the null value of zero appears in the credible interval, similar to using confidence intervals for inference in models estimated with frequentist methods. Instead, the results are interpreted as either “plausibly non-null” (significant) or “found to be null” (non-significant) (81). To fit the models, preliminary psychometric analysis was performed on the three self-regulation scales for both the smoking and BED samples to examine between-time and between-person differences (Supplementary Figure S2). If meaningful, these differences were included in our dynamic mediation models to best tailor the measurement of the self-regulation constructs to each individual person. Models were used to examine the indirect effect of momentary context on health behavior as well as the direct effect of context on health behavior, which was independent of momentary self-regulation.

## Results

### Study participants

#### Binge eating sample

**Figure 1:**
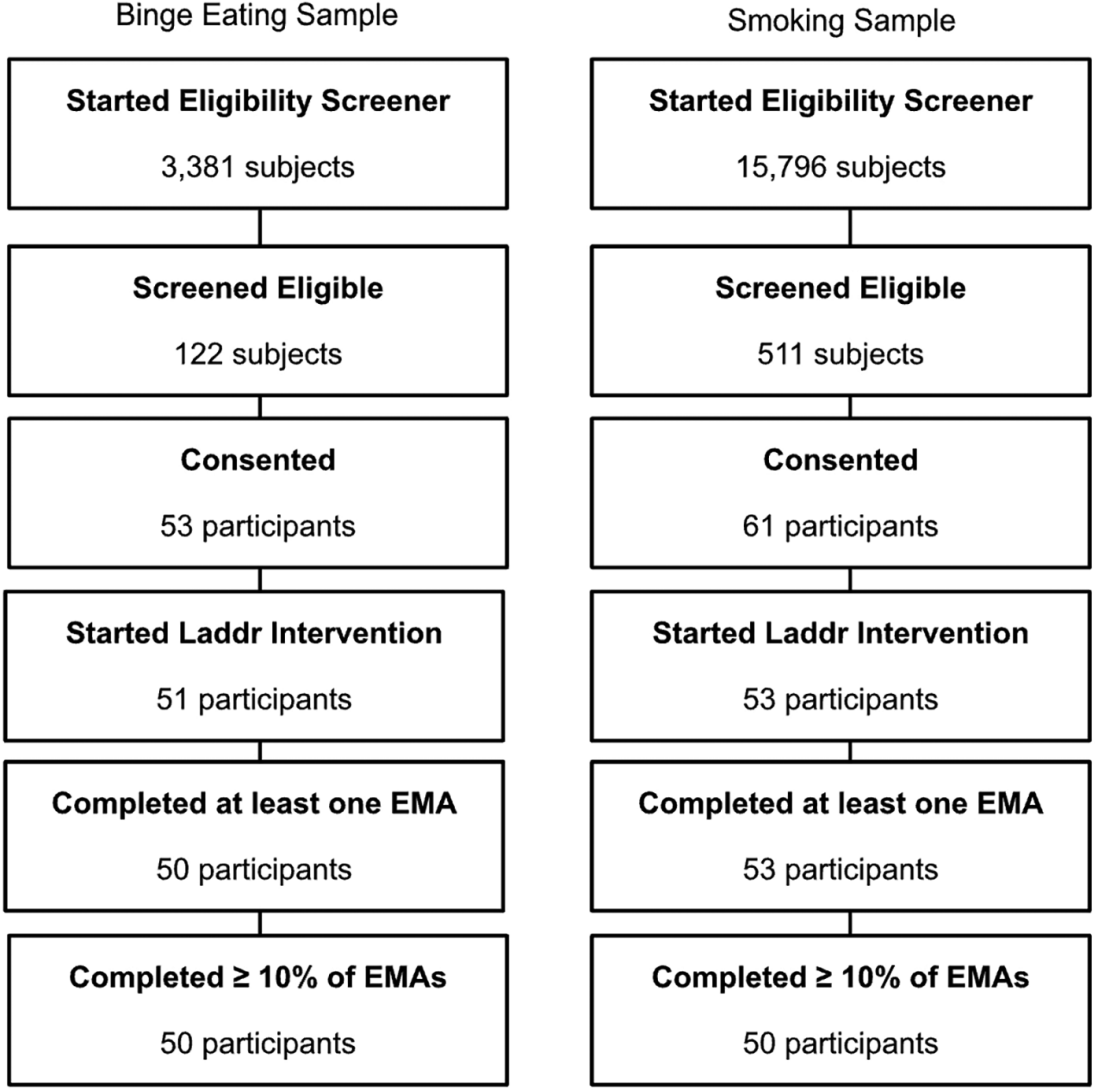
Participant flow chart.

In the BED sample, a total of 3,381 online eligibility screenings were started (Figure 1). Of these individuals, 122 completed the screener and were eligible for the study and 53 of those individuals provided consent. While 52 participants completed the baseline survey and were provided with Laddr subscriptions, 51 participants actively started the Laddr intervention. A total of 50 participants completed at least one EMA and all 50 (100%) had at least 10% of responses over the duration of the 28-day study (≥12 out of 112 total EMAs). Of these 50 participants, 45 participants completed the follow-up surveys (90%). Participants in the BED sample who completed at least 10% of EMAs contributed a total of 4,028 momentary observations with an overall EMA response rate of 72%.

#### Smoking sample

In the smoking sample, a total of 15,796 online eligibility screenings were started (Figure 1). Of these individuals, 511 completed the screener and were eligible for the study and 61 of those individuals provided consent. A total of 55 participants completed the baseline survey and were provided with Laddr subscriptions, and 53 participants actively started the Laddr intervention. These 53 participants also completed at least one EMA and 50 participants (94%) completed at least 10% of responses over the duration of the four-week study (≥12 out of 112 total EMAs). Of the 50 participants who completed at least 10% of EMAs, 43 participants completed the follow-up surveys (86%). Participants in the smoking sample who completed at least 10% of EMAs contributed a total of 3,525 momentary observations with an overall EMA response rate of 63%.

### Baseline characteristics

#### Binge eating sample

The mean age was 34.3 years (SD = 7.8, range = 18–50 years) and mean BMI was 34.0 kg/m^2^ (SD = 5.1, range = 27.4–44.8; Table 1). Most participants were female (58.0%), white (76.0%), non-Hispanic (90.0%), had a bachelor's degree (46.0%) and were married (54.0%). Four (8.0%) participants had been diagnosed with a mental disorder that was not determined to be due to a medical condition or classified as a psychotic disorder. Most participants never smoked (60.0%), while those who had smoked in the past did so for less than one year (55.0%), and all smoked less than 20 cigarettes per day (100.0%; Table 2). Most participants (80.0%) endorsed drinking alcohol in the past year. Among participants who drank alcohol, the highest frequency of drinking was two to four times a week (35.4%), while most participants never engaged in binge drinking (60.0%). Most participants had not smoked cannabis in the past 6 months (77.1%), while the highest frequency among those who did smoke cannabis smoked it weekly (36.4%). Few (20.0%) participants were ever on a diet to lose weight during their life and even fewer (2.0%) had ever been in therapy with a clinician for BED. Of the 50 participants who screened positive for BED on the QEWP-5, the majority reported eating an unusually large quantity of food during a short time period (94.0%), while few participants also reported the use of compensatory behaviors following episodes of binge eating at a frequency only less than once per week required: (1) vomiting to avoid weight gain (4.0%), (2) fasting to avoid weight gain (12.0%), (3) excessive exercise to avoid weight gain (6.0%), and (4) misusing laxatives (2.0%), diuretics (0.0%), or diet pills (0.0%) to avoid weight gain.

**Table 1:** Participant demographics in the combined, smoking, and binge eating samples.

	Combined sample	Binge eating sample	Smoking sample
	*N* = 100	*N* = 50	*N* = 50
Characteristic			
Age, years	34.0 (7.4)	34.3 (7.8)	33.6 (7.1)
Gender			
Female	58 (58%)	29 (58%)	29 (58%)
Male	42 (42%)	21 (42%)	21 (42%)
Race			
Black/African American	10 (10%)	5 (10%)	5 (10%)
White	81 (81%)	38 (76%)	43 (86%)
Asian	6 (6%)	4 (8%)	2 (4%)
Other	3 (3%)	3 (6%)	0 (0%)
Hispanic ethnicity	9 (9%)	5 (10%)	4 (8%)
Body mass index, kg/m^2^	28.9 (6.5)	34.0 (5.1)	23.6 (2.3)
Education			
Some high school or less	1 (1%)	1 (2%)	0 (0%)
High school diploma or GED	15 (15%)	3 (6%)	12 (24%)
Some college or associate's degree	40 (40%)	13 (26%)	27 (54%)
Bachelor's degree	31 (31%)	23 (46%)	8 (16%)
Master's degree	7 (7%)	4 (8%)	3 (6%)
Doctoral degree	6 (6%)	6 (12%)	0 (0%)
Marital status			
Single	31 (31%)	14 (28%)	17 (34%)
Married	46 (46%)	27 (54%)	19 (38%)
Committed Relationship	23 (23%)	9 (18%)	14 (28%)
Mental Health Disorder (Non-medical and non-psychotic etiology)	6 (6%)	4 (8%)	2 (4%)

Note: Categorical or dichotomous variables are reported as *n* (%). Continuous data are reported as mean (SD).

**Table 2:** Participant smoking and binge eating behaviors in the combined, smoking, and binge eating samples.

	Combined sample	Binge Eating Sample	Smoking Sample
	*N* = 100	*N* = 50	*N* = 50
Characteristic			
Duration of smoking (lifetime)			
Never	30 (30%)	30 (60%)	0 (0%)
<1 year	11 (11%)	11 (22%)	0 (0%)
1 year	1 (1%)	0 (0%)	1 (2%)
2 years	1 (1%)	0 (0%)	1 (2%)
3 years	2 (2%)	0 (0%)	2 (4%)
4 years	2 (2%)	1 (2%)	1 (2%)
5–10 years	17 (17%)	6 (12%)	11 (22%)
More than 10 years	36 (36%)	2 (4%)	34 (68%)
Smoking Frequency (current)			
Never	50 (50%)	50 (100%)	0 (0%)
Some days	2 (2%)	0 (0%)	2 (4%)
Every day	48 (48%)	0 (0%)	48 (96%)
Cigarettes smoked per day (current)			
None	50 (30%)	50 (100%)	0 (0%)
<1	0 (0%)	0 (0%)	0 (0%)
1–4	0 (0%)	0 (0%)	0 (0%)
5–9	9 (18%)	0 (0%)	9 (18%)
10–19	28 (28%)	0 (0%)	28 (56%)
20–29	11 (11%)	0 (0%)	11 (22%)
30–39	2 (2%)	0 (0%)	2 (4%)
Fagerström test for nicotine dependence			
Low dependence (score 1–2)	–	–	9 (18%)
Low-to-moderate dependence (score 3–4)	–	–	12 (24%)
Moderate dependence (score 5–7)	–	–	19 (38%)
High dependence (score 8+)	–	–	10 (20%)
Binge Eating Behaviors			
Ever on diet to lose weight	–	10 (20%)	–
Ever been in therapy for binge eating	–	1 (2%)	–
Eating excessive food in short time	–	47 (94%)	–
Vomiting to avoid weight gain	–	2 (4%)	–
Fasting to avoid weight gain	–	6 (12%)	–
Excessive exercise to avoid weight gain	–	3 (6%)	–
Misuse laxatives	–	1 (2%)	–

Note: Categorical or dichotomous variables are reported as *n* (%). Continuous data are reported as mean (SD). The Fagerström Test for Nicotine Dependence was only administered in the smoking sample and the binge eating questionnaire was only administered in the binge eating sample. While some participants in the binge eating sample did report a lifetime prevalence of smoking cigarettes and provided data on duration, frequency, and cigarettes smoked per day above, participants in this sample reported smoking no cigarettes in the past 12 months to be included in the trial. Compensatory binge eating behaviors (e.g., purging, excessive exercise, fasting) were all at a frequency of less than once per week to be included in the binge eating sample.

#### Smoking sample

The mean age was 33.6 years (SD = 7.1, range = 18–49 years) and mean BMI was 23.6 kg/m^2^ (SD = 2.3, range = 18.4–26.8; Table 1). Most participants were female (58.0%), white (86.0%), non-Hispanic (92.0%), and had some college education (54.0%), and 38.0% were married. Two (4.0%) participants had been diagnosed with a mental disorder that was not determined to be due to a medical condition or classified as a psychotic disorder. Most participants smoked more than 10 years (68.0%), smoked every day (96.0%), and smoked between 10 and 19 cigarettes per day (56.0%; Table 2). The mean score on the Fagerström Test for Nicotine Dependence was 5.1 of 10 total (SD = 2.3, range = 1–9). When categorized based on accepted cut-points, 9 (18.0%) participants had low dependence (score 1–2), 12 (24.0%) had low-to-moderate dependence (score 3–4), 19 (38.0%) had moderate dependence (score 5–7), and 10 (20.0%) had high dependence (score 8+). Most participants (70.0%) endorsed drinking alcohol in the past year. Among participants who drank alcohol, the highest frequency of drinking was once monthly or less (32.7%), while about half of participants did endorse engaging in binge drinking (51.4%). Most participants had not smoked cannabis in the past 6 months (73.5%), while most who did smoke cannabis smoked it daily or almost daily (53.9%).

### Models for binge eating sample

#### Perseverance

Perseverance positively predicted Laddr adherence on the following day [*β*_1_ = 0.17, 95% CI = (0.06, 0.45); Table 3]. This coefficient was on the probit scale because adherence was binary, so a one unit increase in perseverance predicted a 0.17 unit increase in the normal latent variable presumed to underlie adherence. Higher Laddr adherence was subsequently a positive predictor of the steps taken on the same day such that those who adhere took about 335 more steps compared to those who did not adhere [*β*_2_ = 0.34, 95% CI = (0.03, 0.72)]. The indirect effect of perseverance on steps taken through Laddr adherence was also positive and plausibly non-null [*β*_1_ × *β*_2_ = 0.05, 95% CI = (0.004, 0.24)]. The conditional direct effect of perseverance on steps taken was found to be null [*β*_3_ = −0.05, 95% CI = (−0.60, 0.45)], suggesting complete mediation. There was a positive feedback loop such that Laddr adherence [*β*_5_ = 0.12, 95% CI = (0.05, 0.26)] and steps taken [*β*_4_ = 0.03, 95% CI = (0.002, 0.07)] both predicted increases in perseverance on the following day.

**Table 3:** Estimates of focal parameters for dynamic mediation models using steps taken as the behavior outcome.

Predictor	Outcome	Notation	Perseverance		Emotion regulation		Mindfulness	
			Est.	CI	Est.	CI	Est.	CI
Self-Regulation, *t* −1	Adherence, *t*	*β*_1_	.**17**	[.06, .45]	.**12**	[.03,.27]	−.03	[−.15, .09]
Adherence, *t* −1	Adherence, *t*	*φ*_2_	.56	[−.07, .81]	−.**44**	[−.69, −.09]	−.36	[−.65, .18]
Adherence *t*	Steps Taken, *t*	*β*_2_	.**34**	[.03, .72]	.**39**	[.08, .74]	.**36**	[.11, .65]
Steps Taken, *t* −1	Steps Taken, *t*	*φ*_3_	−.13	[−.52, .70]	.62	[-.53, .73]	−.**46**	[−.60, −.22]
Steps Taken, *t*	Self-Regulation, *t* + 1	*β*_4_	.**03**	[.00, .07]	-.01	[-.04, .01]	.**03**	[.00, .06]
Adherence, *t*	Self-Regulation, *t* + 1	*β*_5_	.**12**	[.05, .26]	.**16**	[.04, .31]	.**13**	[.02, .24]
Self-Regulation, *t*	Self-Regulation, *t* + 1	*α_φ_*	.35	[−.29, .20]	.**58**	[.47, .68]	.**36**	[.25, .45]
Indirect Effect		*β*_1_ × *β*_2_	.**05**	[.00, .24]	.**04**	[.01, .14]	−.01	[−.07, .03]
Conditional Direct Effect		*β*_3_	−.05	[−.60, .45]	.03	[−.06, .23]	.**57**	[.41, .75]

Note: Bold entries indicate that 0 is not within the 95% credible interval and the effect is non-null (the Bayesian analog of significant).

The Notation column corresponds to the labels from the path diagram. Steps Taken is scaled such that a one-unit change indicates a difference of 1,000 steps. Coefficients from rows with Adherence as the outcome are on a probit scale. Autoregressive effect for self-regulation was modeled as random, so the reported effect is the average across all people.

#### Emotion regulation

Emotion regulation predicted Laddr adherence on the following day [*β*_1_ = 0.12, 95% CI = (0.03, 0.27)], where a one unit increase in emotion regulation predicted a 0.12 unit increase in the normal latent variable presumed to underlie adherence. Higher Laddr adherence was subsequently a positive predictor of steps taken the same day such that adherence predicted an increase of about 389 steps [*β*_2_ = 0.389, 95% CI = (0.080, 0.738)]. The indirect effect of emotion regulation on steps taken through Laddr adherence was also positive and plausibly non-null [*β*_1_ × *β*_2_ = 0.04, 95% CI = (0.01, 0.14)]. The conditional direct effect of emotion regulation on steps taken was found to be null [*β*_3_ = 0.03, 95% CI = (−0.06, 0.23)], suggesting complete mediation through Laddr adherence. There was a positive feedback loop such that Laddr adherence [*β*_5_ = 0.16, 95% CI = (0.04, 0.31)] predicted increases in emotion regulation on the following day, however, steps taken did not predict emotion regulation on the following day [*β*_4_ = −0.01, 95% CI = (−0.04, 0.01)].

#### Mindfulness

Mindfulness did not predict Laddr adherence on the following day [*β*_1_ = −0.03, 95% CI = (−0.15, 0.09)], but Laddr adherence did positively predict the number of steps taken on the same day [*β*_2_ = 0.355, 95% CI = (0.108, 0.648)]. The indirect effect of mindfulness on steps taken through Laddr adherence was found to be null [*β*_1_ × *β*_2_ = −0.01, 95% CI = (−0.07, 0.03)], but the conditional direct effect of mindfulness on steps taken on the following day was positive and plausibly non-null [*β*_3_ = 0.57, 95% CI = (0.41, 0.75)] such that a one standard deviation increase above the person-specific baseline of mindfulness predicted an increase in 570 steps on the following day. There was a positive feedback loop such that Laddr adherence [*β*_5_ = 0.13, 95% CI = (0.02, 0.24)] and steps taken [*β*_4_ = 0.03, 95% CI = (0.00, 0.06)] both predicted increases in mindfulness on the following day.

### Models for smoking sample

#### Perseverance

Perseverance was found to positively predict Laddr adherence on the following day [*β*_1_ = 0.91, 95% CI = (0.60, 1.24)] where a one unit increase in perseverance predicted a 0.91 unit increase in the normal latent variable presumed to underlie adherence (Table 4). However, higher Laddr adherence was not subsequently a predictor of CO values on the same day [*β*_2_ = −0.09, 95% CI = (−0.24, 0.09)]. Consequently, the indirect effect of perseverance on CO values through Laddr adherence was found to be null [*β*_1_ × *β*_2_ = −0.07, 95% CI = (−0.24, 0.08)]. The conditional direct effect of perseverance on CO values on the following day was also found to be null [*β*_3_ = −0.20, 95% CI = (−0.59, 0.10)]. There was a positive feedback loop such that Laddr adherence [*β*_5_ = 0.25, 95% CI = (0.18, 0.35)] and CO values [*β*_4_ = 0.20, 95% CI = (0.09, 0.27)] both predicted increases in perseverance on the following day.

**Table 4:** Estimates of focal parameters for dynamic mediation models using carbon monoxide (CO) samples as the behavior outcome.

Predictor	Outcome	Notation	Perseverance		Emotion regulation		Mindfulness	
			Est.	CI	Est.	CI	Est.	CI
Self-Regulation, *t* −1	Adherence, *t*	*β*_1_	**0**.**91**	[.60, 1.24]	0.00	[−.00, .00]	**−0**.**01**	[−.02, −.01]
Adherence, *t* −1	Adherence, *t*	*φ*_2_	−0.09	[−.19, .03]	**0**.**95**	[.05, .97]	**−0**.**15**	[−.24, −.05]
Adherence *t*	CO, *t*	*β*_2_	−0.09	[−.24, .09]	−0.02	[−.08, .74]	**1**.**14**	[1.04, 1.24]
CO, *t* −1	CO, *t*	*φ*_3_	**−0**.**30**	[−.43, −.19]	−0.49	[−.53, .73]	**0**.**62**	[.57, .67]
CO, *t*	Self-Regulation, *t* + 1	*β*_4_	**0**.**20**	[.09, .27]	−0.04	[−0.27, 4.00]	**2**.**71**	[2.11, 3.42]
Adherence, *t*	Self-Regulation, *t* + 1	*β*_5_	**0**.**25**	[.18, .35]	**−3**.**53**	[−5.62, −2.25]	−8.74	[−10.47, 14.08]
Self-regulation, *t*	Self-Regulation, *t* + 1	*α_φ_*	**0**.**55**	[.46, .62]	**0**.**94**	[.88, 1.00]	**0**.**56**	[.47, .63]
Indirect effect		*β*_1_ × *β*_2_	−0.07	[−.24, .08]	0.00	[.00, .01]	**−0**.**02**	[−.03, −.01]
Conditional direct effect		*β*_3_	−0.20	[−.59, .10]	−0.02	[−.05, .00]	**0**.**06**	[.05, .07]

Note: Bold entries indicate that 0 is not within the 95% credible interval and the effect is non-null (the Bayesian analog of significant). The Notation column corresponds to the labels from the path diagram. Coefficients from rows with Adherence as the outcome are on a probit scale. Autoregressive effect for self-regulation was modeled as random, so the reported effect is the average across all people.

#### Emotion regulation

Emotion regulation did not predict Laddr adherence on the following day [*β*_1_ = 0.00, 95% CI = (−0.00, 0.01)]. Laddr adherence also did not predict CO values on the same day [*β*_2_ = −0.02, 95% CI = (−0.11, 1.14)]. The indirect effect of emotion regulation on CO values was also found to be null [*β*_1_ × *β*_2_ = 0.00, 95% CI = (0.00, 0.01)]. The conditional direct effect of perseverance on CO values on the following day was also found to be null [*β*_3_ = −0.02, 95% CI = (−0.05, 0.00)]. There was a negative feedback loop such that Laddr adherence [*β*_5_ = −3.53, 95% CI = (−5.62, −2.25)] predicted decreases in emotion regulation on the following day, however, CO values did not predict emotion regulation on the following day [*β*_4_ = −0.04, 95% CI = (−0.27, 4.00)].

#### Mindfulness

Mindfulness was found to negatively predict Laddr adherence on the following day [*β*_1_ = −0.01, 95% CI = (−0.02, −0.005)] where a one unit increase in mindfulness predicted a 0.01 unit decrease in the normal latent variable presumed to underlie adherence. Higher Laddr adherence was related to higher CO values on the same day [*β*_2_ = 1.14, 95% CI = (1.04, 1.24)]. The indirect effect of mindfulness on CO values through Laddr adherence was negative and plausibly non-null [*β*_1_ × *β*_2_ = −0.015, 95% CI = (−0.03, −0.01)], indicating the possibility of a suppression effect. The conditional direct effect of mindfulness on CO values on the following day was positive and plausibly non-null [*β*_3_ = 0.06, 95% CI = (0.05, 0.07)]. There was a positive feedback loop such that CO values predicted increases in mindfulness on the following day [*β*_4_ = 2.71, 95% CI = (2.11, 3.42)], but Laddr adherence did not predict mindfulness on the following day [*β*_5_ = −8.74, 95% CI = (−10.47, 14.08)].

## Discussion

To our knowledge, this is the first study to examine momentary self-regulation as a behavior change mechanism across different health risk behaviors and to explore how adherence to a digital therapeutic can mediate the relationship between self-regulation and these risk behaviors. While other studies have found that lapses in self-regulation are related to increased smoking, physical inactivity, and binge eating, among other risky behaviors, there has been little evaluation of the mechanisms behind these relationships, and especially the dynamic nature of these mechanistic processes in daily life (23, 29, 31, 82). The present study explored these dynamic mechanisms in real-life situations.

First, we hypothesized that several components of momentary self-regulation would predict adherence to the Laddr intervention on the following day in both samples and found that higher perseverance increased adherence to the intervention across both people with obesity/overweight and BED and people who smoke regularly. Next, we hypothesized that treatment adherence would predict the objective behavioral health outcomes and found that higher adherence increased physical activity levels among people with obesity/overweight and BED. Lastly, we hypothesized that treatment adherence and the behavioral outcomes would act as a positive feedback loop to predict self-regulation at the next measurement. We found that both adherence to the intervention and the behavioral outcomes (steps taken and CO values) predicted several components of increased momentary self-regulation across both samples, although results were more mixed in the smoking sample.

### Momentary self-regulation impacted adherence to a digital therapeutic

While self-regulation is usually examined as a static trait-level characteristic, this study utilized a novel momentary self-regulation scale developed by our interdisciplinary team to measure self-regulation across time and contexts (33, 52). Recent studies suggest that self-regulation is a dynamic process that changes based on individual contexts, but, to our knowledge, this metric is the first assessment tool to examine the dynamic nature of self-regulation (33). This scale was embedded within a novel digital therapeutic called Laddr and successfully administered through mobile devices to assess momentary self-regulation in a nonlaboratory, naturalistic setting (52). As also suggested by prior studies, our results demonstrate that self-regulation has variability depending on real-world internal and external contexts in two exemplar groups of people with health risk behaviors (33, 52).

This current study utilized the novel momentary self-regulation scale to examine if momentary self-regulation impacted adherence to a digital health intervention. It is critically important to understand the mechanisms underlying effective health behavior change and determine how these mechanisms may differ across different health conditions (83). Here, “mechanism” refers to intervention-induced changes in psychological, behavioral, or biological factors, which then cause changes in health risk behaviors (84).

Results suggest that putative targets of momentary self-regulation did impact adherence to the digital therapeutic across groups of people with different health risk behaviors. The perseverance target of momentary self-regulation was the only singular target found to positively predict Laddr adherence on the following day in both people with obesity/overweight and BED and people who smoke regularly. This suggests that higher goal setting, tracking, and continual focus on achieving goals aimed at increased levels of physical activity or decreased levels of smoking, respectively—all key components of perseverance—may be related to future adherence to a digital health intervention on the following day for both of these health risk behaviors (52). Additionally, the emotion regulation target of momentary self-regulation was found to positively predict Laddr adherence on the following day in people with obesity/overweight and BED. This suggests that effectively managing and responding to emotional experiences may also be related to future adherence to a digital health intervention on the following day, particularly in people with obesity/overweight and BED (52).

### Targeting self-regulation through a digital therapeutic

Next, we examined if increasing self-regulation through a digital therapeutic then increased adherence to the intervention and increased pro-health behaviors. This was another highly innovative aspect of the present study. Indeed, there are over 300,000 health-related smartphone applications on both the Apple and Google Play stores, with over 10,000 claiming to influence behavioral health (85). Most of these interventions are uploaded to the store without evaluation with scientific methods, and even fewer have examined underlying mechanisms of their claimed behavior change (85).

This is in stark contrast to the drug discovery process, which includes strict guidelines, including early drug discovery, a pre-clinical phase of research, clinical phases of research, and regulatory approval, often over numerous years of testing (86). These studies examine the effect of the drug on a biological target (e.g., a receptor, enzyme, protein, gene) and must be performed across numerous assays, doses, and populations as applicable (87). The goal of this process is to identify a biological target, and not solely to develop a treatment with clinical benefit. In contrast, most studies of behavior change interventions only examine specific clinical endpoints like smoking or obesity and do not routinely examine these putative targets or the mechanisms of behavior change (51).

This study utilized the experimental medicine approach, proposed by the NIH's Science of Behavior Change (SOBC) initiative and demonstrated that targeting self-regulatory targets through a digital health intervention impacted adherence to the intervention and increased pro-health behaviors. Higher Laddr adherence was found to mediate the relationship between the perseverance and emotion regulation targets of momentary self-regulation and the number of steps taken in people with obesity/overweight and BED. This suggests that adherence to Laddr increased the behavioral health outcome of physical activity in this sample. Thus, this study demonstrated that engaging the self-regulatory targets of perseverance and emotion regulation produced a desired change in medical regimen adherence and in health behavioral outcomes in people with obesity/overweight and BED.

### Health behavior outcomes between the binge eating and smoking samples

Although numerous interventions have been effective in initiating and maintaining health behavior change and medical regimen adherence, including those targeting smoking and binge eating, most of this work has been siloed (50, 88–90). That is, most interventions target only one disease or disorder at a time, while the need to reduce health risk behaviors is ubiquitous across numerous clinical populations. This prompted us to examine these mechanisms in two exemplar samples within this work, people with obesity/overweight and BED and people who smoke regularly, to transdiagnostically study momentary self-regulation across different health-risk behaviors.

That said, the relationships between self-regulation, adherence to Laddr and pro-health behaviors were more mixed in the smoking sample compared to the BED sample. One possible reason for this may be the specific medical regimen—an exclusively digital behavioral intervention—offered to the participants in this study. That is, numerous other studies have suggested that BED and obesity can be effectively managed with behavioral health treatments. Specifically, cognitive behavior therapy (CBT) has repeatedly been shown to be effective in managing BED (91). The Laddr intervention incorporated CBT-based self-monitoring, control strategies, activity goals, and problem solving to help participants develop regular, moderate eating patterns, as web-based, self-help strategies have been shown in previous studies to be efficacious with decreased average number of binges (91).

However, behavioral interventions alone have not always been as efficacious for quitting smoking compared to combined behavioral and pharmacological interventions across other health conditions (92–94). Pharmacotherapies are often a critical part of effective smoking cessation interventions to treat nicotine dependence. Multiple systematic reviews have found that combining both behavioral therapy and pharmacotherapy is more effective in promoting smoking abstinence compared to either strategy alone (95–97). Most clinical trials examining behavioral support for smoking cessation also include a pharmacotherapy arm, and a recent meta-analysis of over 23,000 participants in 65 studies found that combined treatment was associated with 15% higher rates of smoking abstinence (95).

Although the present study did not exclude individuals taking nicotine replacement therapy, use of other medications for smoking cessation (e.g., bupropion, varenicline) was exclusionary. And prior work has demonstrated the value of these medications as part of smoking cessation treatment. For example, a randomized controlled trial of varenicline and CBT vs. placebo medication and CBT for smoking cessation found that 60% of participants in the varenicline group and only 19% in the placebo group had smoking abstinence at 1 year follow-up (93, 94). Together, these studies and our exclusion of participants with concurrent pharmacotherapy for smoking cessation might suggest why adherence to the Laddr behavioral health intervention demonstrated increased pro-health behaviors in the BED sample compared to the smoking sample. Future studies examining digital therapeutics in people who smoke regularly should also add a digital intervention-plus-pharmacotherapy arm to further examine the impact of combining these two interventions on promoting smoking cessation.

### Understanding the role of mindfulness in promoting adherence and health behavior change

The mindfulness factor of momentary self-regulation also had mixed results in the BED and smoking samples. Mindfulness alone did not predict greater Laddr adherence on the following day in the BED sample and predicted less adherence on the following day within the smoking sample. One possible explanation of this finding may be because mindfulness is “the act of paying attention in the present moment,” as opposed to future orientation (98). People practicing mindfulness try to self-regulate their attention to the current moment and this may direct their attention away from behaviors that promote future medical regimen adherence (99). Similar to our findings, a recent systematic review examining mindfulness interventions to improve medication adherence across different medical conditions found mixed results with only 44% of the included studies reporting increased medication adherence with mindfulness training (88). The review concluded that the use of mindfulness interventions to promote medication adherence remains understudied across different diseases and our current study also supports the need for future research on the impact of momentary mindfulness on medical regimen adherence (88).

When examining the health behavior outcome in the smoking sample, mindfulness directly predicted higher CO values on the following day, but also indirectly predicted lower CO values on the following day through Laddr adherence. This finding may suggest that higher mindfulness, possibly focused on smoking abstinence, on the preceding day may be related to increased smoking as a compensatory mechanism on the following day. Nicotine compensation is a behavior related to biological dependence of nicotine where people who smoke regularly increase their number of cigarettes, puffs, or depth of the inhalation in response to a period of abstinence from nicotine (100). Laboratory studies have shown that short-term nicotine restriction may lead to compensatory smoking and increased levels of CO, which might help explain why mindfulness predicted higher CO values on the following day in our study (101). But continued adherence to Laddr may disrupt that relationship by providing more tools to individuals to help them effectively reduce smoking behavior.

Lastly, it is important to recognize the possible implications of these results to the mindfulness subscale itself, which may be measuring a distinct construct from other measures of momentary self-regulation. Several studies have described mindfulness as a unique self-regulation strategy that differs from other emotion regulation strategies (102–105). Additionally, the actual neural mechanisms behind mindfulness-based regulation may differ between participants, where some participants may use cognitive reappraisal strategies based on top-down neural systems, and other participants engage in more emotion regulation based on bottom-up neural systems (106, 107).

### Transdiagnostic mediating effects of momentary self-regulation

Overall, our series of studies was able to accomplish the three initiatives of the NIH SOBC initiative, including (1) investigating mechanisms of behavior change across different health behaviors, (2) finding common predictable targets of health behaviors, and (3) modifying clinical endpoints through replicable and scalable behavioral health interventions (51). First, our interdisciplinary team studied the ontology of self-regulatory processes in people with obesity/overweight and BED and people who smoke regularly. These are two exemplar samples where “lapses” in self-regulation can contribute to reduced medical regimen adherence and negative health outcomes (1, 2). Second, three momentary self-regulation targets of perseverance, emotion regulation, and mindfulness were examined as common predictable targets across health risk behaviors of smoking and physical inactivity (33). Third, adherence to the Laddr digital therapeutic was examined as a predictor of the clinical endpoints: number of steps taken for physical inactivity and CO values for smoking.

Our studies applied novel technologies and methods to inform the ontology of momentary self-regulation in people with obesity/overweight and BED and people who smoke regularly. Our work complements and extends the important work of four other NIH SOBC-funded studies which examined self-regulation in the context of prediabetes, mental and physical health, mood and weight, and health behaviors in children (108). Three additional SOBC-funded studies examined other key factors known to impact health behavior change, including (1) stress reactivity and stress resilience and (2) interpersonal and social processes (51). All these projects utilized the experimental medicine approach to perform assay development and validation of targets on one of these three domains: self-regulation, stress, and social processes (108). While these studies allowed for the comparison of different behavior change targets, future studies could examine the combined influence of these factors on how self-regulation, stress, and social processes work together to impact health behavior change. While studying these domains is important in understanding the putative targets of health behavior change, it is also important to recognize that there are many factors that can impact behavior change beyond those included in the SOBC initiative (109). More recently, the SOBC program now hosts a resource and coordinating center, including the Checklist for Investigating Mechanisms in Behavior-change Research (CLIMBR) guidelines, designed to support the use of the experimental medicine approach in the experimental study of other malleable behavioral processes (109).

Future studies could also incorporate additional passive sensing data, such as smartphone geospatial data, which can allow for the examination of external contexts on an individual's self-regulation and adherence to a digital health intervention. Passive sensing can incorporate different streams of smartphone data including physiology, movement, location, audio, and smartphone app usage to predict current and future changes in health behavior (110–112). Such passive sensing data have predicted future behavioral outcomes, including, for example, momentary changes in depressed mood in people with depression, and have been studied across other mental health problems, sleep disorders, substance use, stress, and cardiovascular disease (112, 113). While our study included measures of momentary self-regulation collected through EMAs, future studies can also include smartphone passive sensing to determine how external contexts affect an individual's self-regulation and adherence to digital health interventions.

### Limitations

This study had several limitations. First, while we examined two exemplar samples of people with BED and people who smoke, the inclusion criteria prevented overlapping samples. Therefore, this study may not be generalizable to people who smoke and have co-occurring BED and therefore not generalizable to people with multiple health risk behaviors. Second, while this study included a racially diverse sample comparable to the 2022 United States census, individuals identifying as Hispanic or Latino were underrepresented in this sample. This suggests that targeted sampling strategies need to do better at recruiting based on ethnicity within these groups of people with health risk behaviors. Third, our online screening procedure did not initially prohibit prospective participants from completing the screening questionnaire more than once, so it is possible that some participants may have been included in the study who should have been deemed ineligible. We implemented guardrails against repeated screening attempts in the later stages of recruitment by manually reviewing the screening questionnaires for identifiers (e.g., name, phone number, ZIP Code) prior to enrolling participants. Participants who enrolled before we implemented these additional checks would have remained in the study. Fourth, the dynamic mediation models only included three self-regulation scales (perseverance, emotion regulation, mindfulness) and excluded the sensation seeking scale. Psychometric analysis indicated that the items of sensation seeking did not represent a single construct in the sample, so it may be considered inappropriate to model these items as one construct like the other self-regulation scales. Because an objective of this study was to examine mechanisms across different targets of momentary self-regulation, we determined that modeling sensation seeking in a different way than the other three scales would make the results less generalizable and harder to compare between targets. Our prior study examining putative targets of momentary self-regulation did not find any significant associations between urge to smoke or urge to binge eat and momentary sensation seeking (33). This suggests that sensation seeking might play a different role than the other targets of momentary self-regulation in both the smoking and BED samples. Although our momentary self-regulation scale had strong construct and predictive validity and intra- and interindividual variability, future research should further explore the sensation seeking items. Lastly, the current study was an observational study of self-regulation targets, medical regimen adherence, and health behavior outcomes, so other unmeasured confounding variables may have contributed to these observed relations. However, the longitudinal nature of the study reduces the influence of potential confounders. Future directions of this research include evaluating effects of randomization to the Laddr intervention or comparison conditions and applications to other momentary behaviors.

## Conclusions

This study provides evidence that a digital therapeutic targeting self-regulation can modify several relationships between momentary self-regulation, medical regimen adherence and behavioral health outcomes in people with health risk behaviors. Results demonstrate that the perseverance target of momentary self-regulation increased Laddr medical regimen adherence on the following day in both people with obesity/overweight and BED and people who smoke regularly. Then, higher adherence to the Laddr intervention predicted the behavioral health outcome of increased physical activity. Additionally, higher Laddr adherence then predicted increased perseverance and emotion regulation targets on the following day people with obesity/overweight and BED. Together this suggests that the influence of momentary self-regulation on health risk behaviors may occur through increased medical regimen adherence to the Laddr digital health intervention, particularly in people with obesity/overweight and BED. Future studies should validate these findings across various other digital health interventions and other health risk behaviors. Within the larger NIH SOBC initiative, this work demonstrated the ability to assess several transdiagnostic mediating effects of momentary self-regulation on medical regimen adherence and pro-health behavioral outcomes.

## Data Availability

The raw data supporting the conclusions of this article will be made available by the authors, without undue reservation.

## Supplementary material

Supplementary Figure S1 The within-person structural model portion of the dynamic mediation model. Self-regulation is a latent variable that predicts (latent-centered) behavior and treatment adherence at the next time-point. Treatment adherence then predicts behavior at the same time-point. Both treatment adherence and behavior then predict self-regulation as the next time-point. Lagged effects of self-regulation and the amplitude of the self-regulation time-series are permitted to vary across people.

Supplementary Figure S2 The between-person portion of the dynamic mediation model. Each random parameter from the within-person model is featured as a latent variable with a fixed effect, between-person variance, and possible covariances with other latent variables. The residual variance capturing the amplitude of the time-series is modeled on the natural log scale to ensure that values are strictly positive.
