# Supplementary figures and images for: Adherence to a digital therapeutic mediates the relationship between momentary self-regulation and health risk behaviors
Source: Front Digit Health. 2025 Feb 4;7:1467772. doi: 10.3389/fdgth.2025.1467772 (PMC11841403; doi:10.3389/fdgth.2025.1467772)

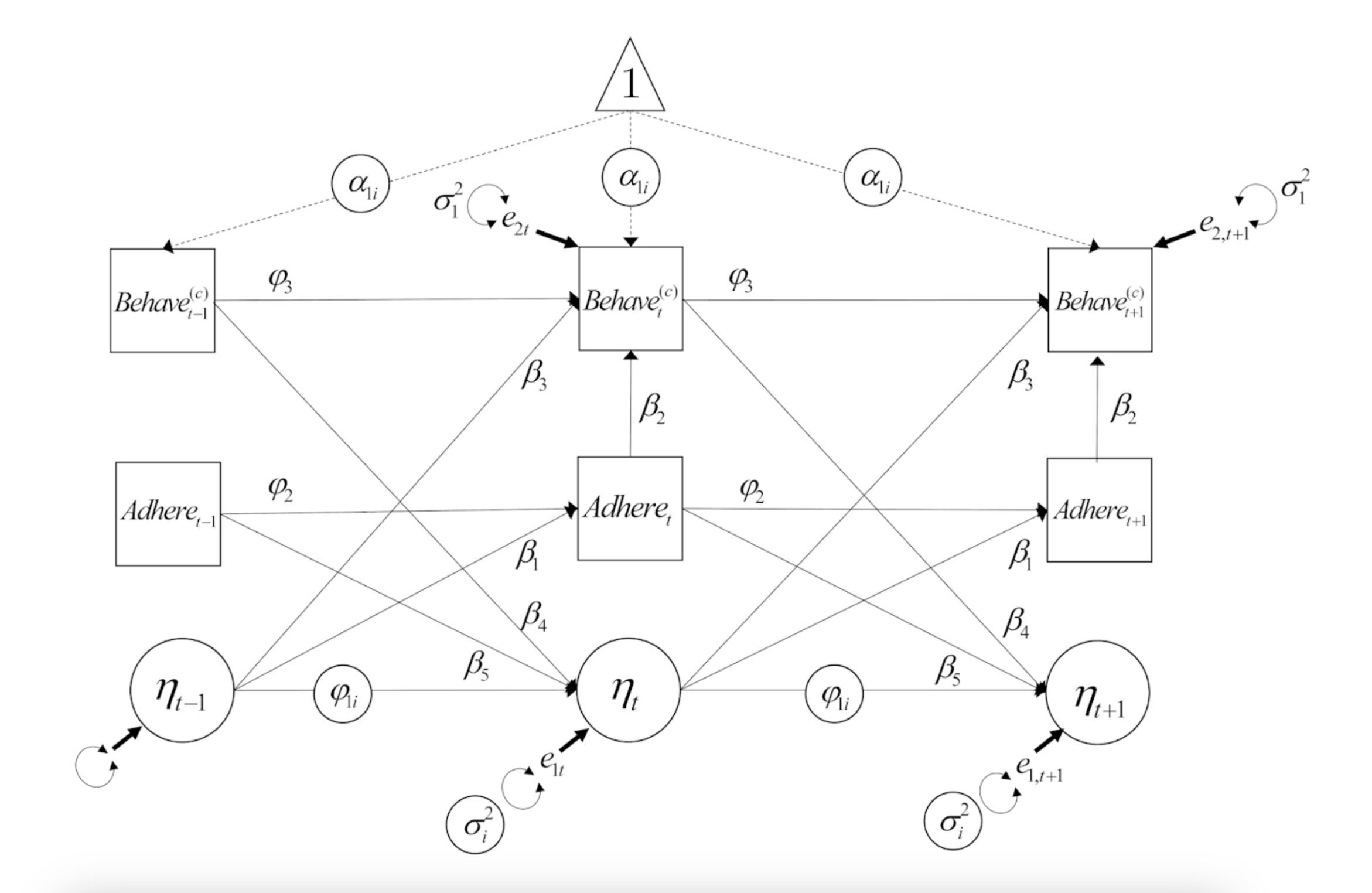

Supplement: Supplementary Figure S1 — The within-person structural model portion of the dynamic mediation model. Self-regulation is a latent variable that predicts (latent-centered) behavior and treatment adherence at the next time-point. Treatment adherence then predicts behavior at the same time-point. Both treatment adherence and behavior then predict self-regulation as the next time-point. Lagged effects of self-regulation and the amplitude of the self-regulation time-series are permitted to vary across people. [file Image1.jpeg]

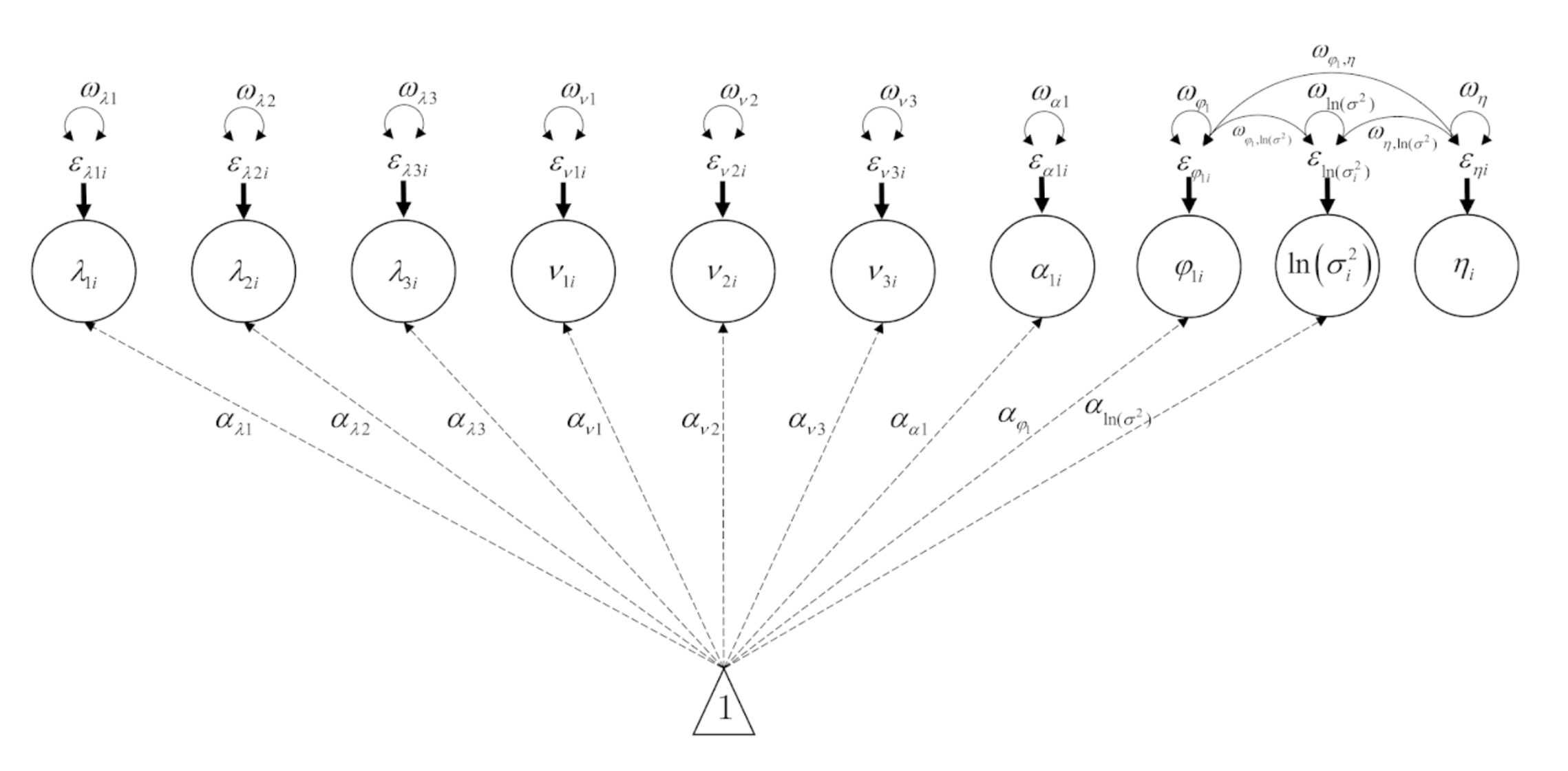

Supplement: Supplementary Figure S2 — The between-person portion of the dynamic mediation model. Each random parameter from the within-person model is featured as a latent variable with a fixed effect, between-person variance, and possible covariances with other latent variables. The residual variance capturing the amplitude of the time-series is modeled on the natural log scale to ensure that values are strictly positive. [file Image2.jpeg]
