# Supplementary material for: Adherence to a digital therapeutic mediates the relationship between momentary self-regulation and health risk behaviors
Source: Front Digit Health. 2025 Feb 4;7:1467772. doi: 10.3389/fdgth.2025.1467772 (PMC11841403; doi:10.3389/fdgth.2025.1467772)
Supplement: Supplementary file 3 [file Table1.docx]

| **Table S1.** Results for cross-classified factor analysis to assess approximate measurement invariance over time and people | | | | | | | | | | | | | |  |
| --- | --- | --- | --- | --- | --- | --- | --- | --- | --- | --- | --- | --- | --- | --- |
|  |  |  | Between-Person | | | |  | Between-Time | | | | | |  |
|  |  | Loading  Mean | Threshold Variance | Threshold Variance CI | Loading Variance | Loading Variance CI |  | Threshold Variance | Threshold Variance CI | | Loading Variance | | Loading Variance CI | |
| Perseverance |  |  |  |  |  |  |  |  |  |  | |  | |  |
|  | Item 1 | .73 | .05 | [.01, .09] | .08 | [.05, .13] |  | .00 | [.00, .00] | .00 | | [.00, .00] | |  |
|  | Item 2 | .78 | .14 | [.08, .23] | .07 | [.04, .11] |  | .00 | [.00, .00] | .00 | | [.00, .00] | |  |
|  | Item 3 | .58 | .23 | [.13, .36] | .08 | [.05, .12] |  | .00 | [.00, .01] | .00 | | [.00, .00] | |  |
| Emotion Regulation |  |  |  |  |  |  |  |  |  |  | |  | |  |
|  | Item 1 | 1.08 | .54 | [.30, 1.03] | .88 | [.54, 1.51] |  | .01 | [.00, .03] | .00 | | [.00, .02] | |  |
|  | Item 2 | 4.06 | .33 | [.02, 1.38] | 4.27 | [2.60, 7.39] |  | .01 | [.00, .06] | .01 | | [.00, .08] | |  |
|  | Item 3 | 1.81 | .46 | [.26, .84] | 1.04 | [.62, 1.82] |  | .01 | [.00, .03] | .00 | | [.00, .03] | |  |
| Mindfulness |  |  |  |  |  |  |  |  |  |  | |  | |  |
|  | Item 1 | 1.54 | .18 | [.07, .39] | .54 | [.32, .94] |  | .01 | [.00, .03] | .00 | | [.00, .01] | |  |
|  | Item 2 | 1.72 | .04 | [.00, .14] | .83 | [.48, 1.45] |  | .03 | [.00, .06] | .00 | | [.00, .02] | |  |
|  | Item 3 | 3.34 | .39 | [.05, 1.11] | 3.62 | [2.00, 6.50] |  | .02 | [.00, .07] | .01 | | [.00, .04] | |  |

Note: The Perseverance items are modeled as continuous and items from all other scales are modeled as categorical with a probit link. Loading means are interpreted as regression coefficients for the Perseverance scale but item discriminations for all other scales.
